# Enhanced Ferromagnetism in Nanoscale GaN:Mn Wires Grown on GaN Ridges

**DOI:** 10.3390/ma10050483

**Published:** 2017-05-02

**Authors:** Ji Cheng, Shengxiang Jiang, Yan Zhang, Zhijian Yang, Cunda Wang, Tongjun Yu, Guoyi Zhang

**Affiliations:** 1 Research Center for Wide Gap Semiconductors, State Key Laboratory for Artificial Microstructure and Microscopic Physics, School of Physics, Peking University, Beijing 100871, China; masterlaoji@gmail.com (J.C.); jshx1989123@163.com (S.J.); zjyang@pku.edu.cn (Z.Y.); cdwang44@126.com (C.W.); 2 Institute of Condensed Matter Physics, School of Physics, Peking University, Beijing 100871, China; zhang_yan@pku.edu.cn

**Keywords:** semiconductor, GaN:Mn, magnetic, MOCVD

## Abstract

The problem of weak magnetism has hindered the application of magnetic semiconductors since their invention, and on the other hand, the magnetic mechanism of GaN-based magnetic semiconductors has been the focus of long-standing debate. In this work, nanoscale GaN:Mn wires were grown on the top of GaN ridges by metalorganic chemical vapor deposition (MOCVD), and the superconducting quantum interference device (SQUID) magnetometer shows that its ferromagnetism is greatly enhanced. Secondary ion mass spectrometry (SIMS) and energy dispersive spectroscopy (EDS) reveal an obvious increase of Mn composition in the nanowire part, and transmission electron microscopy (TEM) and EDS mapping results further indicate the correlation between the abundant stacking faults (SFs) and high Mn doping. When further combined with the micro-Raman results, the magnetism in GaN:Mn might be related not only to Mn concentration, but also to some kinds of built-in defects introduced together with the Mn doping or the SFs.

## 1. Introduction

Magnetic semiconductors have attracted extensive attention in the past decade due to their potential applications for spintronics [1,2]. A high Curie temperature (Tc)—considered as crucial to making useful spintronic devices a reality—has been predicted for Mn-doped wide-bandgap GaN (GaN:Mn) semiconductors [3]. However, in this regard, there is still a great diversity in views according to the reported literature [3,4,5,6,7,8,9,10,11,12,13,14,15,16]. Bonanni et al. grew GaN:Mn with a very low Curie temperature (Tc), which is a dilute magnetic insulator with Mn^3+^ ions substitutionally incorporated in the host GaN crystal, and they successfully applied several theoretical approaches to explain the results [8,9,10]. On the other hand, there are many other groups growing GaN:Mn with very high Tc, both in the forms of conventional film [11,12] and nanostructure [13,14], and also with the methods of molecular-beam epitaxy (MBE) [15] and metalorganic chemical vapor deposition (MOCVD) [16]. The results cannot be unified in the structure of one theory. In general, though the origin of GaN:Mn ferromagnetism is still debated, it has been commonly agreed upon that a higher Mn percentage leads to a higher Tc in GaN:Mn. As for the experimental part, the doping rate is usually low for the low solubility of Mn; thus, the saturated magnetization of GaN:Mn—even with room temperature ferromagnetism—is still relatively small [17,18]. Moreover, the mechanism of the magnetism in GaN:Mn is still not fully understood [19,20], which in turn hinders the applications of the material.

Semiconductor nanostructures in low-dimension are good subjects for both studies on the physics of the material and potential applications in devices [21,22,23]. With no strict demand for nanoscale-patterned substrate or growth conditions, epitaxy growth on ridges is a popular way to form nanoscale wires [24]. Moreover, it is reported that the unique stress field distribution at the ridge top favors further Mn doping [25], thus providing an approach toward enhanced magnetism.

In this work, nanoscale GaN:Mn wires were fabricated on the top of GaN ridges by MOCVD. The Mn content in the wires reached a notable 1.31%, and the ferromagnetism was enhanced by two orders of magnitude compared with the conventional GaN:Mn film. A large number of stacking faults (SFs) were observed in the wires by transmission electron microscopy (TEM), and they showed a correlation with the Mn doping distribution. When further combined with the micro-Raman results, the magnetism in GaN:Mn might be related not only to Mn concentration but also to some types of built-in defects introduced together with the Mn doping or the SFs.

## 2. Materials and Methods

**Figure 1:**
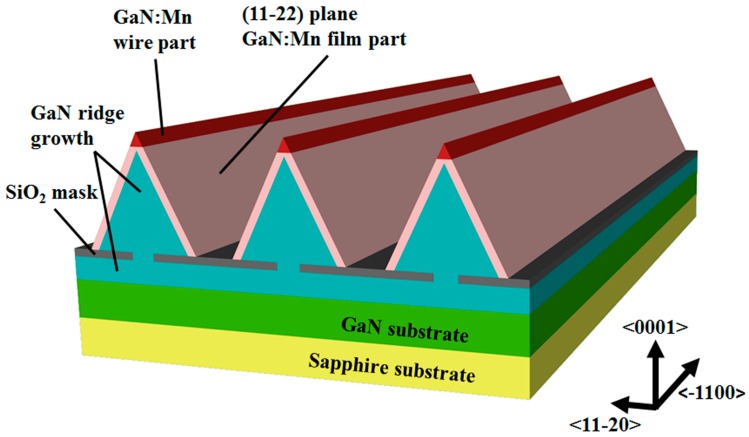
The schematic structure of the GaN:Mn wires grown on GaN ridges.

Referring to Figure 1, we started with a 3 μm GaN film grown on sapphire and deposited a 500 nm-thick SiO_2_ mask layer at 250 °C by plasma-enhanced chemical vapor deposition (PECVD). The surface of the GaN was then patterned by SiO_2_ into periodic stripes with 16.5 μm-wide masked areas and 3.5 μm-wide openings along the <–1100> direction using ultraviolet photolithography and inductively-coupled plasma (ICP) dry etching. 

Then, the stripe-patterned substrate was put into a MOCVD system with trimethyl-gallium (TMGa), bis-(monomethyl-cyclopentadienyl) manganese ((MCP)_2_Mn), and ammonia (NH_3_) gases used as the source materials for Ga, Mn, and N, respectively. Eight micrometer-wide and 7 μm-high triangular cross-section GaN ridges were grown with 136 μmol/min TMGa, 8 slm NH_3_ in a close-coupled showerhead reactor MOCVD system. The substrate temperature was 1040 °C, and the reactor pressure was 200 mbar. Finally, the GaN:Mn layer (about 200 nm thick) was grown under 11.3 μmol/min TMGa, 0.87 μmol/min (MCP)_2_Mn, and 4.8 slm NH_3_ in a low-pressure horizontal reactor MOCVD system. The substrate temperature was 1060 °C, and the reactor pressure was 100 mbar. Finally, two kinds of epitaxial GaN:Mn were obtained on the GaN ridges shaped along the <–1100> direction: a 200 nm-thick film part on the (11-22) plane sidewalls, and a 400 nm diameter wire part on the ridge top.

For comparison, the same process was carried out, but with the mask opening along <11-20> direction. Due to the rapid lateral growth, the ridge top could not combine into a wire. Therefore, a sample with no GaN:Mn wire, but only the (1-10-1) plane GaN:Mn film on the sidewalls and (0001) plane GaN:Mn film on the top, were obtained (not shown in the figure). In addition, a reference sample of a 200 nm conventional c-plane GaN:Mn film was also grown on the GaN film with the same growth conditions.

## 3. Results and Discussion

### 3.1. Content Analysis

**Figure 2:**
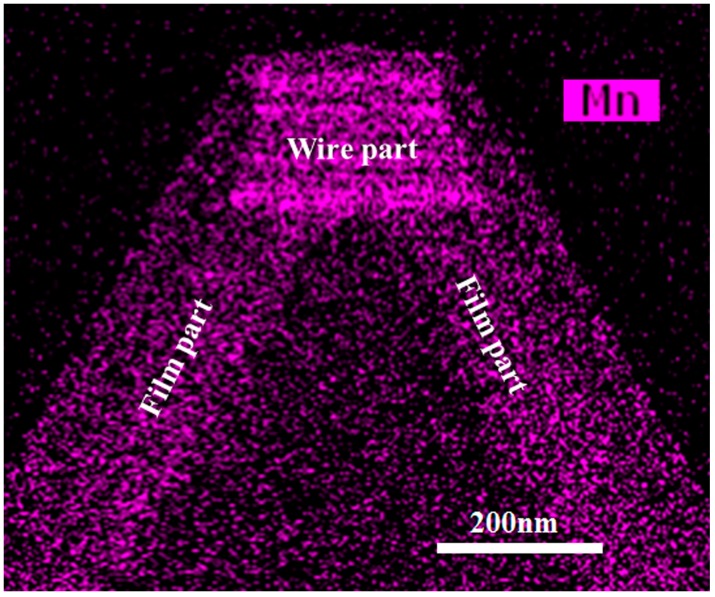
The cross-section energy dispersive spectroscopy (EDS) mapping image of the upper part of one ridge.

A cross-section energy dispersive spectroscopy (EDS) mapping (in an FEI Osiris TF-20 field emission gun (FEG)/TEM, FEI, Hillsboro, OR, USA) for Mn elements in Figure 2 gives a clear picture of our sample: a wire part of higher Mn content on the top of the ridge and a film part with relatively less Mn composition on the two sides of the ridge. The Mn signal can be seen in both the wire part and the film part, showing successful Mn doping growth. The brighter signal in the wire part indicates Mn content as high as 1.31%, while the darker signal for the film part was too weak to give a reliable Mn content (under 1%, the instrument’s detection limit).

Secondary ion mass spectrometry (SIMS) was also applied as a supplementary measure to confirm the content. We focused the ion beam on the wire part and film part, and obtained the content data, respectively. Consistent with the EDS results, the Mn concentration was detected as ~4 × 10^20^ cm^−3^ in the wire part and ~1 × 10^20^ cm^−3^ in the film part. In addition, the SIMS data showed the same level of Mn concentration in the film part, the (1-10-1) plane GaN:Mn film, and the conventional c-plane GaN:Mn film. 

### 3.2. Magnetic Properties

Hysteresis loops were measured at 300 K in a commercial superconducting quantum interference device (SQUID) magnetometer MPMS XL from Quantum Design (Quantum Design Inc., San Diego, CA, USA). As the magnetic signals for most magnetic semiconductors—including GaN:Mn—are usually weak, a very careful operation and a measurement as sensitive as possible was performed in the reciprocating sample option (RSO) mode to obtain reliable data (this is a trivial measuring process, but for samples with very weak magnetism, we should take care with the magnetic contaminants from air, tweezers, etc., and ensure the instrument is set up properly. Interested readers are referred to [26] for further details).

**Figure 3:**
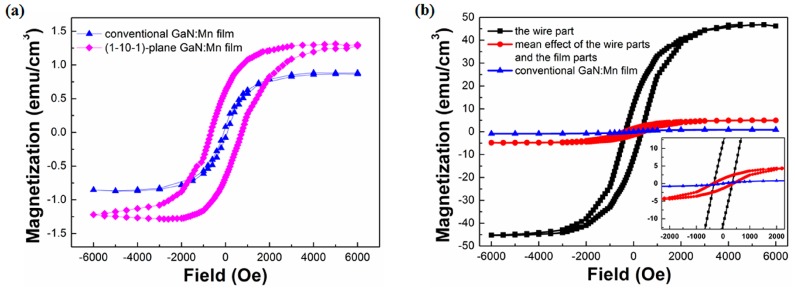
(**a**) Hysteresis loops of the (1-10-1) plane GaN:Mn film and the conventional GaN:Mn film at 300 K; and (**b**) hysteresis loops of the GaN:Mn wire part, the overall mean effect of the film part and the wire part, and the conventional GaN:Mn film at 300 K. Inset: an enlarged image of the zero field part of the hysteresis loops.

The overall mean effect of the film parts and the wire parts can be evaluated from the SQUID magnetic signal. T film part grown on a (11-22) plane is an epitaxial film on a semi-polar plane, just like the situation of the (1-10-1) plane GaN:Mn film [27]. Moreover, a similar amount of Mn has been doped based on the SIMS results described above. It is thus proper that the film part has the same level of magnetism as the (1-10-1) plane GaN:Mn film. Moreover, as shown in Figure 3a, the magnetism of the (1-10-1) plane GaN:Mn film was at the same level as the conventional GaN:Mn film. Therefore, it was safe to regard the film part as a conventional GaN:Mn film, approximately, and the hysteresis loop purely of the wire part could be drawn as shown in Figure 3b, with the hysteresis loop of the mean effect of the film parts (red dot) and the wire parts (black square) as a reference. 

According to the SQUID results in Figure 3b, the wire part showed (compared with the conventional GaN:Mn film) an increment of two orders of magnitude, from 0.86 emu/cm^3^ to 46 emu/cm^3^ (4.8 emu/cm^3^ for the overall mean effect of the film parts and the wire parts) in magnetization saturation. From an enlarged image of the zero field part (inset of Figure 3b), the residual magnetization showed a significant increment from 0.08 emu/cm^3^ to 11 emu/cm^3^ (1.5 emu/cm^3^ for the overall mean effect of the film parts and the wire parts), and the coercivity greatly increased from 80 Oe to 350 Oe (380 Oe for the overall mean effect of the film parts and the wire parts), which was notably much larger than their nano counterparts of about 35 Oe [7,13].

### 3.3. Structural Analysis

**Figure 4:**
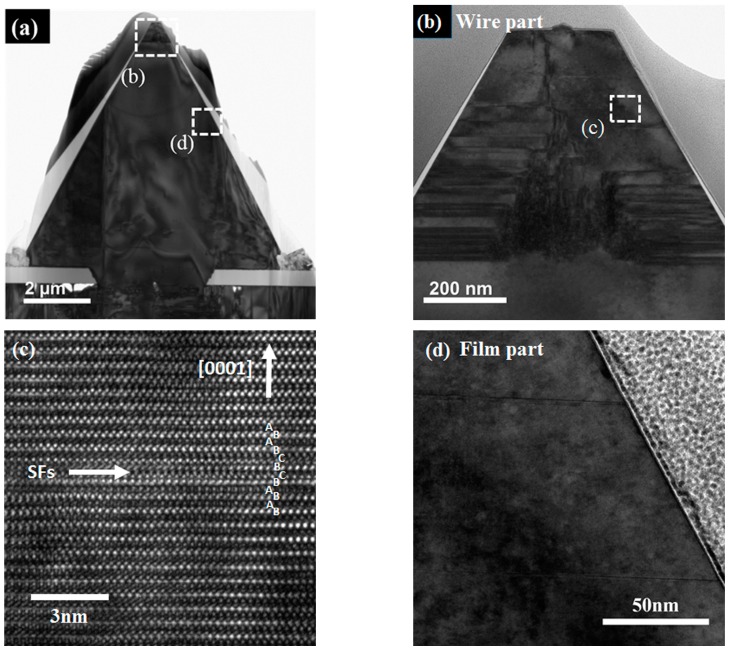
(**a**) Bright-field TEM image of the cross-section of a whole ridge. Enlarged high-resolution TEM (HR-TEM) image of (**b**) the wire part with (**c**) an enlarged image for a stacking fault (SF) and (**d**) the film part.

To understand the reason for the high Mn concentration, cross-section TEM (FEI Osiris TF-20 FEG/TEM) was used to observe the structure of the sample. The TEM-ready sample was perpendicular to the ridge using an in situ focused ion beam (FIB) lift-out technique on an FEI dual-beam FIB/SEM, and the results are shown in Figure 4. 

High-resolution TEM (HR-TEM) images demonstrate a good crystallinity, but a few SFs in the film part are shown in Figure 4d. As for the wire part, a large number of SFs can be seen in Figure 4b,c. These SFs were considered to be related to stress (also indicated in the micro-Raman results explored later), induced by the fast c-axis growth and perturbation from the Mn presentation during the growth [28]. Moreover, with a closer look into the wire part in the EDS mapping results in Figure 2, we could find that the brighter wire part actually consisted of a large number of horizontal lines which just corresponded to the SF lines in Figure 4b. As the brighter horizontal lines in the EDS mapping image result according to the locally higher Mn concentration lines, the increase of Mn concentration in the wire part could therefore be related to the SFs, and the reason behind this might well be the decreasing forming energy near the fault planes [29,30].

### 3.4. Micro-Raman Spectra

**Figure 5:**
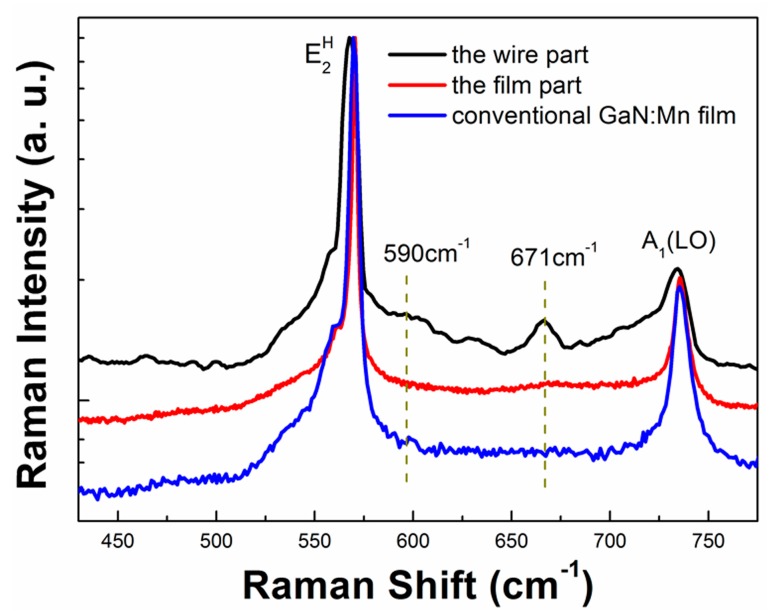
Micro-Raman spectra of the wire part, the film part, and the conventional GaN:Mn film.

As the two orders of magnitude increment in magnetism of the wire part was relatively large in response to the approximately four times increment in Mn concentration, it is difficult to believe that all of the enhancement in the magnetism could be attributed to the increase of the Mn doping rate. Thus, micro-Raman spectroscopy (with an Invia 6365 Confocal Raman microscope, Gloucestershire, UK) was applied to study the correlations among Mn content, structural features, and magnetism in the wire part. The result is shown in Figure 5, and the spectra of the film part and the conventional GaN:Mn film are also drawn for comparison. 

In contrast to the result of the conventional GaN:Mn film, the E_2_^H^ phonon mode at 570 cm^−1^ shifted to a lower frequency in the wire part, indicating a release of the compressive stress in the wire part, which could be explained by the growth rate change in different facets at the ridge top [31,32,33,34]. The Mn-N pair-related 590 cm^−1^ peak [35] could be seen only in the wire part, and not detected in the film part or the conventional GaN:Mn film, indicating a high Mn concentration in the wire part, which is consistent with the Mn concentration results from EDS and SIMS. Notably, there was a significant 671 cm^−1^ peak in the wire part, while this peak was not so obvious in the film part or the conventional GaN:Mn film. This mode was assigned to disorderly-activated Raman scattering of built-in defects in the intrinsic GaN lattice, such as vacancies-related defects, which can be induced with Mn doping or the SFs, which furthered the Mn doping [36,37]. Moreover, there is a strong asymmetric broadening towards the lower frequency of the A_1_(LO) peak near 734 cm^−1^. This asymmetric broadening of the A_1_(LO) peak has been also observed in (Ga,Al)As materials, and is explained by the spatial correlation model [38]. For the situation of GaN:Mn in the wire part, more lattice disorder and other built-in defects may be induced with greater Mn incorporation. These lattice disorders and built-in defects disrupt the long-range ionic ordering in GaN, thus weakening the electric field associated with the LO mode, and thus lowering its frequency. All of the results showed a correlation between the built-in defects and the ferromagnetic enhancement in the wire part, and thus supported the viewpoint that aside from the Mn doping rate, the built-in defects might play an important role in the magnetism of GaN:Mn [39,40,41,42].

From a general perspective, when magnetic atoms are doped into a semiconductor, the magnetism would of course be increasing in accordance with the doping rate. However, in this nanoscale GaN:Mn material, the magnetism notably increased two orders of magnitude while the Mn doping increased only about four times, which greatly highlights the effect—besides the doping rate—of defects, for example. Our result may provide a new route to understanding the long-debated problem of GaN:Mn magnetism; that is, a comprehensive study on defects and doping in nanoscale GaN:Mn as an experimental material base.

## 4. Conclusions 

In this work, nanoscale GaN:Mn wires with 1.31% Mn doping were grown on the top of a ridge of GaN by MOCVD. While there was only an increment in Mn content of about four times compared with conventional GaN:Mn film, the ferromagnetism was enhanced by two orders of magnitude. A large number of SFs were observed in the wire part, which was correlated with the higher Mn doping rate there. In addition, the micro-Raman results gave a hint that the significant ferromagnetism enhancement may be due to the built-in defects introduced, together with the Mn doping or the SFs. To summarize, the magnetism in GaN:Mn might be related not only to Mn concentration, but also to some types of built-in defects.
